# Effectiveness of Mouth Washes on Streptococci in Plaque around Orthodontic Appliances

**DOI:** 10.5402/2011/954053

**Published:** 2010-09-21

**Authors:** Behnam Khosravani Fard, Mahmood Ghasemi, Hossein Rastgariyan, Seyed Hadi Sajjadi, Houshang Emami, Masoomeh Amani, Mohammad Hosein Kalantar Motamedi

**Affiliations:** ^1^Departments of Orthodontics, Periodontics, and Surgery, Azad University, Pasdaran St, Tehran, Iran; ^2^Trauma Research Center, Baqiyatallah University, Vanak Square, MollaSadra Ave., Tehran, Iran

## Abstract

*Background and Purpose*. Fixed orthodontics may be associated with accumulation of Mutans Streptococci (MS), enamel demineralization, and an increased number of carious lesions, predominantly in sites adjacent to brackets. This study was undertaken to assess the effectiveness of Listerine, Oral-B, and Ortho-kin on the accumulation of MS in plaque around orthodontic brackets. *Materials and Methods* A double-blind randomized cross-over clinical trial on 25 orthodontic patients, classified into 6 groups was done to assess MS in plaque and saliva with the side specific modified Strip-Mutans technique and the plaque (PI) was measured before and after rinsing using 3 types of commercial mouth-rinses. A washout period (3 weeks) was awaited between using each mouth-rinse and the data was analyzed via Wilcoxon and Kruskal Wallis statistical tests. *Results*. This study of 25 patients, 5 men and 20 women, with an average age of 19 ± 6/3 assessed the effectiveness of mouth-rinses on MS. Our results showed that Ortho-kin had a better effect than Oral-B and Listerine (*P* < 0/09). Ortho-kin also had better effects than Oral-B and Listerine on plaque accumulation (*P* < 0/001). *Conclusion*. Ortho-kin showed better effects on decreasing MS and PI because it contained chlorhexidine.

## 1. Introduction

Orthodontic treatments may induce oral ecologic changes, leading to increase of Streptococcus mutans in saliva and plaque [1, 2]. Orthodontic brackets play a significant role in gathering microbial plaque [3, 4]. Caries-preventive measures, good oral hygiene, noncariogenic diet, and regular fluoride supplementation are often insufficient in preventing the occurrence of new carious lesions in orthodontic patients with high caries activity [5, 6]. Also, it has been shown that orthodontic treatment with fixed appliances results in enamel demineralization and increased numbers of carious lesions, predominantly in sites adjacent to brackets [7]. Preventive efforts in these risk groups have been focused on direct suppression of the cariogenic microflora by chemotherapeutics as an adjunct to improved oral hygiene. Chlorhexidine is a potent documented antimicrobial agent against streptococci and dental caries [8–10]. It has been suggested that applying chlorhexidine in the form of varnish reduces the number of MS in plaque and saliva for 4 weeks, but this effect has been tested on teeth without orthodontic appliances. On the other hand, studies performed on high-risk orthodontic patients with highly concentrated varnish treatment, has not demonstrated to influence caries reduction [11, 12]. Another mouthrinse, which has clinically-proven effectiveness in decreasing microbial plaques, is Listerine. Studies have shown that Listerine effectively decreases formation of microbial plaque and gingivitis [13, 14]. Studies on microbiological effectiveness of Listerine showed that Listerinecan not affect the structure of microbial plaque [15]. Yet, no studies regarding Listerine, Ortho-kin, and Oral-B have been found evaluating this issue. In this study, we addressed the clinical effectiveness of Ortho-kin, Listerine, and Oral-B on Mutans Streptococcus (MS) existing in plaque around the orthodontic brackets and in saliva, and also on decreasing dental plaque (PI).

## 2. Materials and Methods

A double-blind randomized cross-over clinical trial on 25 orthodontic patients, classified into 6 groups was done to assess MS in plaque and saliva with the side specific modified Strip-Mutans technique and the plaque (PI) was measured before and after using 3 types of commercial mouthrinses. A washout period (3 weeks) was awaited between using each mouthrinse. The data included clinical exam, inspection, and microscopic observation techniques. Patients with fixed orthodontic appliances, inserted at least 2 months prior to the start of the study were chosen agreeing with the terms and conditions and completing the information sheets. Conditions of termination of this study were use of antibiotics, illness, treatment with topical fluoride, use of mouthrinse in the last month, and dental caries. After selecting the patients, they were provided with the instructions about oral hygiene prior to the study. The plaque MS scores were determined with the side-specific modified Strip-Mutans as originally described by Wallman and Krasse [16] and modified by Twetman [17]. Selected teeth for plaque sampling were isolated with cotton rolls and dried, and then, samples were carefully taken with a sterile curette on the sites around the brackets of teeth 15, 25, 35, and 45. All brackets had been placed by the same orthodontist with orthophosphoric acid 37% (Swiss Coltene), and glass ionomer Fuji (Ortho L.C, G.C.). Sampled plaque was immediately spread on the roughened side of the plastic strip with square tip from the Strip-Mutans kit (Orion Diagnostica, Strip-Mutans, Finland). Additionally, a saliva Strip-Mutans test was performed and evaluated on each participant. The strips were allowed to dry for 5 minutes at room temperature and were then incubated for 96 hours in a liquid medium. The composition of the medium was similar to the composition of Mitis Salivarius Agar, with a sucrose concentration increased to 30%. Addition of a Bacitracin disc from the kit, resulted in a final concentration of 0.36 U/CC of Bacitracin per mL of medium. After 96 hours of incubation in the liquid medium, the scores of MS in plaque and saliva were recorded with the aid of a stereo-microscope with 10–25x magnification. The number of colony-forming units (CFU) with characteristic morphology was screened and scored on the scale of 0–3.

### 2.1. Score Allocation

Scores were allocated as follows:

- 0 indicates no CFU (MS below detection level);
- 1 indicates 1–10 CFU, corresponding to approximately <10^4^–10^5^ CFU;
- 2 indicates 10–100 CFU, corresponding to approximately =10^5^–10^6^ CFU;
- 3 indicates >100 CFU, corresponding to >10^6^ CFU.

In clinical exams, the dimensions of dental plaque were measured by disclosing solution (Dentsply, USA). All dental surfaces were painted over with solution using a brush. Surfaces of teeth with plaque, were colored by this solution. Then, these surfaces were numerated and the dimension of PI was recorded. Afterwards, the patients were classified into 6 groups and 3 mouthrinses, Ortho-kin (Kin company Spain), Listerine (Warner-Lambert company USA) and Oral-B (P&G company England), prepared in white pet glasses and filled and coded by a third person, were applied to these groups. The patients used these mouthrinses in each group according to rules below:

  Code 1:    group 1 1   2   3 *n* = 4
  Oral-B      group 2 1   3   2 *n* = 5
  Code 2:    group 3 2   1   3 *n* = 4
  Listerine   group 4 2   3   1 *n* = 4
  Code 3:    group 5 3   1   2 *n* = 5
  Ortho-kin  group 6 3   2   1 *n* = 3
            
$\overset{⃡}{3\;\text{weeks}}\;\;\;\;\;\overset{⃡}{3\;\text{weeks}}$
         (Wash out period)

Each mouthrinse was used for 3 weeks. After this period, patients waited a 3-week wash out period (without using any mouthrinse) for liquidation of mouthrinse effectiveness and the dimension of MS returning to baseline values again. At the end of 3 weeks, the dimensions of MS existing in plaque and saliva and also the dimension of PI was measured and recorded.

## 3. Patient Instructions

It was requested that the patients use each of these 3 mouthrinses in the morning and evening after brushing their teeth and 15 mL for 30 seconds to rinse the mouth and then, not to eat or drink for 30 minutes. Ill patients or those who used drugs, or for any reason did not follow the instructions, were eliminated from the study. The data was analyzed via Wilcoxon and Kruskal Wallis statistical tests.

## 4. Results

This study on 25 patients, 5 men and 20 women, with an average age of 19 ± 6/3 assessed the effectiveness of mouthrinses on MS. Plaque sampling from each patient measured on 4 teeth (100 samples), surveyed 300 teeth because the design of research was a cross-over for 3 mouthrinses. Our results showed that Ortho-kin had a better effect than Oral-B and Listerine (*P* < 0/09). Ortho-kin also had better effects than Oral-B and Listerine on plaque accumulation (*P* < 0/001). The dimension of changes in accumulation of MS on each tooth according to kind of mouthrinse is shown in Table 1. It shows better effectiveness of Ortho-kin (*P* < .09). Ortho-kin was 14% better than Listerine (*P* < .04).

**Figure 1:**
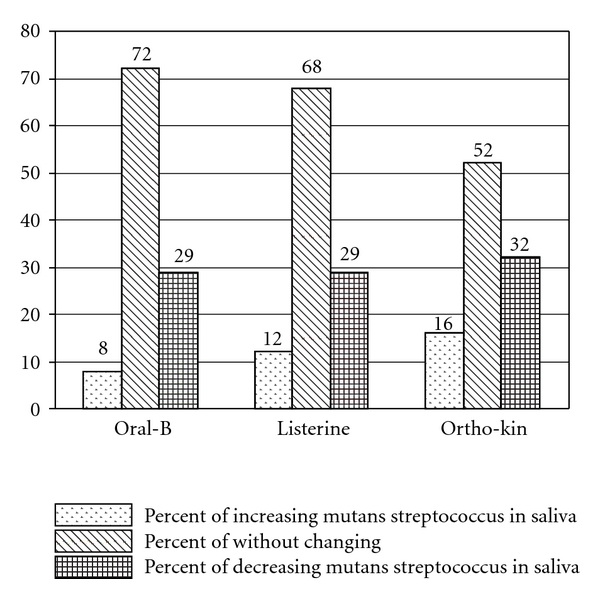
Distribution of the percentage of Streptococcus mutans changes in saliva relevant to the kind of mouthrinse.

The dimension of MS changes in person's saliva given according to the kind of mouthrinse in Figure 1, shows that the dimension of changes, percentage of increase, without change, and decrease of Streptococcus mutans in saliva in 3 groups were similar and without statistical differences (*P* < .4).

The amount of microbial plaque in the subjects and the changes according to the kind of mouthrinse are shown in Table 2. It shows that Oral-B (about 10.9 ± 5.61 or 20.7 percent), Listerine (about 12.2 ± 9.76 or 24 percent), and Ortho-kin (about 14.5 ± 9.99 or 31.7 percent) caused reduction of dental plaque, with significant statistical differences in each group (*P* < .01).

## 5. Discussion

In this study, the effectiveness of 3 mouthrinses, Ortho-kin (with combination of Chlorhexidine de gluconate, Sodium Fluoride, Zinc Acetate), and Oral-B (with combination of Methyl Paraben, Cetyl Pyridinium Chloride, Sodium Fluoride, Propyl Paraben, Alcohol), and Listerine (with combination of Tymol, Eucalyptol, Methyl Salicylate, and Menthol) was assessed on accretion of Streptococci mutans existing in plaque and saliva and the amount of PI.

The results showed that in decreasing the accretion of Streptococci mutans existing in plaque around orthodontic brackets, Ortho-kin was better than Oral-B and Listerine. In several studies, it has been shown that following treatment with highly concentrated CHX, Streptococci mutans can be suppressed effectively for a prolonged period of time [18–20]. Also in a study by Maltz on subjects who were not orthodontic patients, it was shown that CHX was effective in decreasing Streptococci colonization and also in decreasing dental caries [10, 21].

It should be noted that studies performed in high-risk orthodontic patients did not demonstrate any significant differences in caries after repeated application of high or low concentrated CHX varnishes [1, 5]. Although chlorhexidine therapy, prior to or during orthodontic treatment has been shown to significantly reduce colonization of Streptococci mutans it had no effect on caries activity [1, 2].

The duration of Streptococci suppression, partly depends on the extent to which retention niches are coated with CHX. The presence of bands and orthodontic brackets prevents the effective function of CHX in all areas with Streptococci mutans [22–25]. The results of our study showed that all three mouthrinses were similar in their effect on Streptococci in saliva, without significant statistical differences. Also, Ortho-kin with CHX could not decrease the Streptococci in saliva and could not act better than Listerine or Oral-B.

In another study, a layer of polyurethane sealant was placed over the chlorhexidine containing layer to retain it on the teeth. Since the polyurethane sealant also slows the loss of chlorhexidine into the saliva, it causes increased effectiveness on saliva MS without increase in the concentration of CHX [26]. Listerine, well known as an antiseptic since the last century, did not show any bactericidal effects in Brecx et al. study [12]. This is in accordance with the results of Siegrist et al., who found no significant reduction in the number of bacteria in dental plaque formation in subjects using Listerine as compared to a placebo. In our study, Listerine was not effective on accumulation of MS in plaque or saliva and Ortho-kin was more effective than Listerine and Oral-B in decreasing PI.

## 6. Conclusion

Ortho-kin showed better effects on decreasing MS and PI because it contained chlorhexidine.

## Figures and Tables

**Table 1:** Distribution of teeth surveyed with respect to MS accumulation based on the kind of mouthrinse.

Kind of mouthrinse	Decrease	Without change or increase	Total
Oral-B	23	67	100
Listerine	30	70	100
Ortho-kin	44	46	100

**Table 2:** Distribution of subjects under orthodontic therapy with regard to microbial plaque changes based on the kind of mouthrinse.

PI mouthrinse	Before using mouthrinse	After using mouthrinse	Changes	Result (Wilcoxon Test)
Oral-B	52.7 ± 17.2	45.9 ± 18.7	10.9 ± 5.61	*P* < .01
Listerine	50.7 ± 17.7	48.2 ± 18.7	12.2 ± 9.79	*P* < .01
Ortho-Kin	45.8 ± 15.05	44.1 ± 12.2	14.5 ± 9.99	*P* < .001
Result (Kruskal Wallis test)	*P* < .2	*P* < .8	*P* < .2	
